# Supplementary material for: Structures reveal how the Cas1-2/3 integrase captures, delivers, and integrates foreign DNA into CRISPR loci
Source: bioRxiv. 2025 Jun 11:2025.06.10.658980. Preprint. [Version 1] doi: 10.1101/2025.06.10.658980 (PMC12259127; doi:10.1101/2025.06.10.658980)
Supplement: 1 [file NIHPP2025.06.10.658980V1-supplement-1.pdf]

bioRxiv preprint doi: https://doi.org/10.1101/2025.06.10.658980; this version posted June 11, 2025. The copyright holder for this preprint (which was not certified by peer review) is the author/funder, who has granted bioRxiv a license to display the preprint in perpetuity. It is made available under aCC-BY 4.0 International license.

SUPPLEMENTAL FIGURES

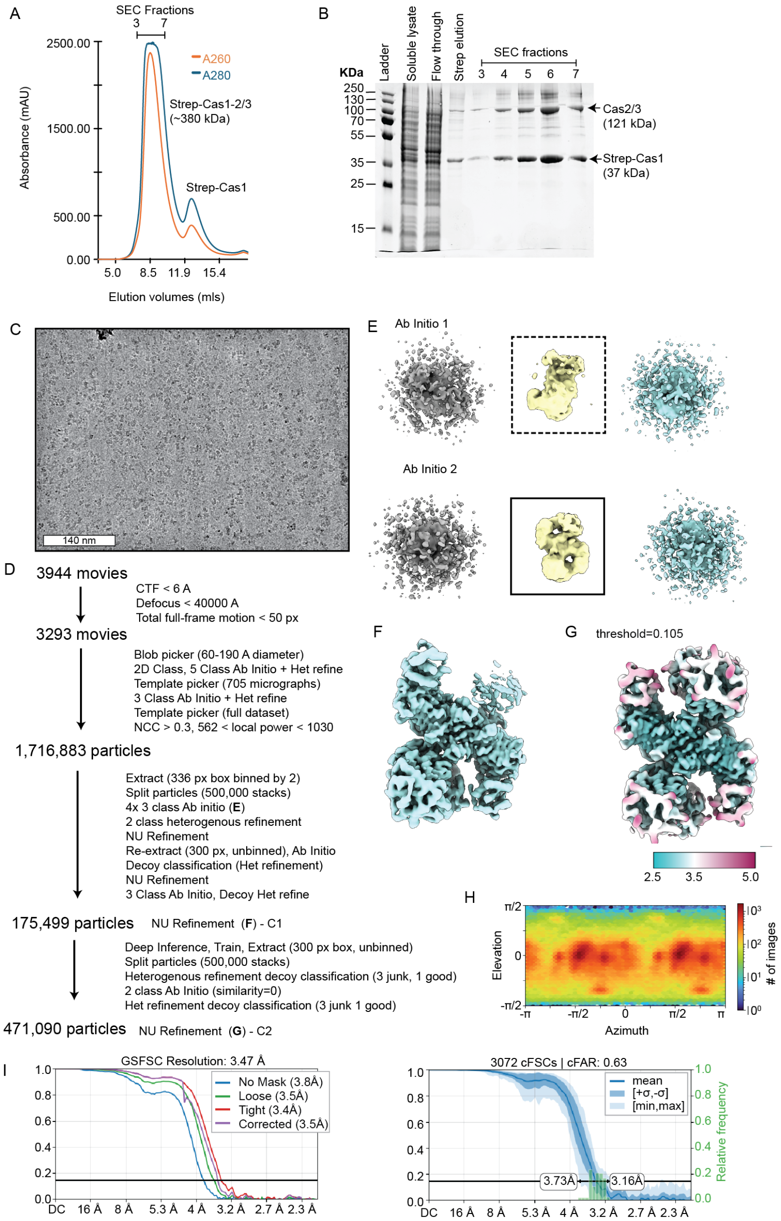

Figure S1. Purification and data processing of the Cas1-2/3 capture complex. Related to Figure 1 (A) Size exclusion chromatography (SEC) of Cas1-2/3 results in a monodispersed complex with an estimated molecular weight of 380 kDa. (B) SDS-PAGE of the fractions following affinity purification, and size exclusion. (C) Sample micrograph at a nominal magnification of 81,000. (D) Data processing summary. (E) Results from two of four total, 3-class *Ab initio* reconstructions and heterogenous refinements provided two volumes (boxed) for baited heterogeneous refinement (dashed box indicates volume with severe anisotropy, solid box indicates a volume with well-distributed views). (F) Non-uniform (NU) refinement of 175,499 particles used to train cryoSPARC's Deep Inference job. (G) Final NU refinement of 471,090 particles reconstructed with C2 symmetry imposed (threshold = 0.105). Coloring by local resolution (estimation at FSC 0.5) reveals most of the complex is resolved below 3.5 Å. (H) Azimuth plot from cryoSPARC of the final C2 NU refinement. (I) FSC and conical FSC plots of the final C2 NU refinement.

bioRxiv preprint doi: https://doi.org/10.1101/2025.06.10.658980; this version posted June 11, 2025. The copyright holder for this preprint (which was not certified by peer review) is the author/funder, who has granted bioRxiv a license to display the preprint in perpetuity. It is made available under aCC-BY 4.0 International license.

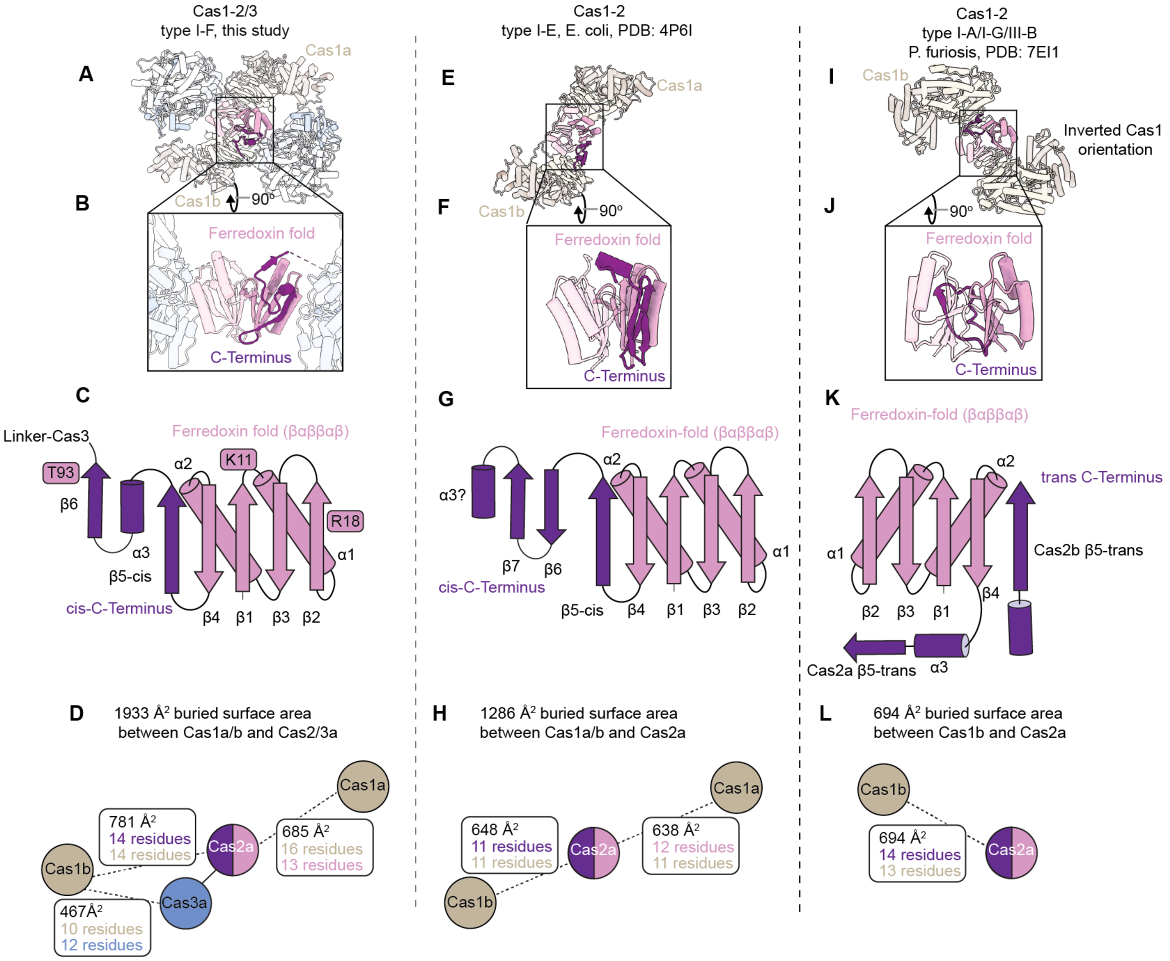

Figure S2. The C-terminal topology of Cas2 determines the Cas1 interface. Related to Figure 1 (A, E, I). The variable Cas2 C-terminus determines the identity and extent of partner interaction. Structures of experimentally determined Cas1-2 complexes from the PDB shown at 50% transparency, with one subunit of the Cas2 dimer colored to show the core ferredoxin-like fold (pink) and the variable C-terminus (purple). (B, F, J) Cas2-containing subunit only, rotated 90 degrees to visualize the dimer interface. (C, G, K) Ribbon diagrams for each Cas2 structure in panels B, F, J. (D, H, L) Interaction diagrams depicting the subunit interactions for a single Cas2 dimer, with the number of residues and buried surface area of each interface depicted.

bioRxiv preprint doi: https://doi.org/10.1101/2025.06.10.658980; this version posted June 11, 2025. The copyright holder for this preprint (which was not certified by peer review) is the author/funder, who has granted bioRxiv a license to display the preprint in perpetuity. It is made available under aCC-BY 4.0 International license.

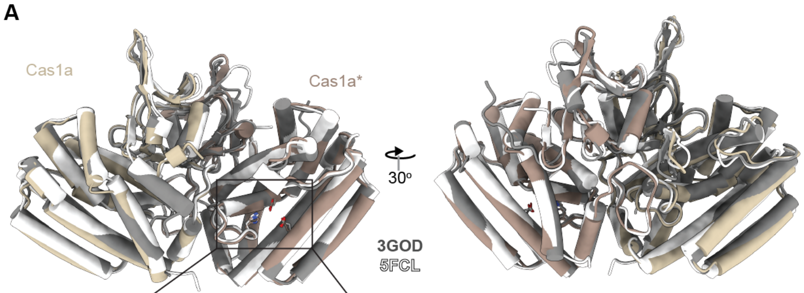

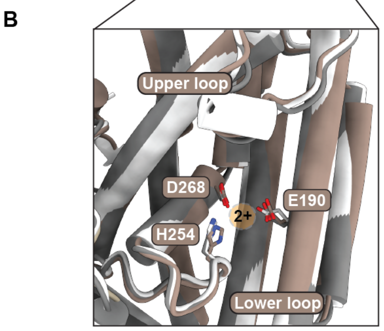

C

	3GOD	5FCL
RMSD	0.787 Å	0.743 Å
Pruned Cα	278	288
RMSD (all)	1.785 Å	1.689 Å
All Cα	296	310

Figure S3. Cas2/3 association does not change Cas1 dimer architecture. **Related to Figure 1** (A) Structural overlay of previously determined Cas1 dimer crystal structures (PDB: 3GOD, *P. aeruginosa* in grey, PDB: 5FCL, *P. atrosepticum* in white) onto one Cas1 dimer from the Cas1-2/3 complex (colored as in Fig 1). (B) Close-up of the Cas1* active site shows nearly perfect overlap of active site residues involved in coordinating a divalent metal ion (not observed in the cryo-EM structure due to EDTA in the purification buffer) (C) Table of root mean square deviations across pruned and unpruned Cα atoms, from the matchmaker (mmaker) command in UCSF ChimeraX.

bioRxiv preprint doi: https://doi.org/10.1101/2025.06.10.658980; this version posted June 11, 2025. The copyright holder for this preprint (which was not certified by peer review) is the author/funder, who has granted bioRxiv a license to display the preprint in perpetuity. It is made available under aCC-BY 4.0 International license.

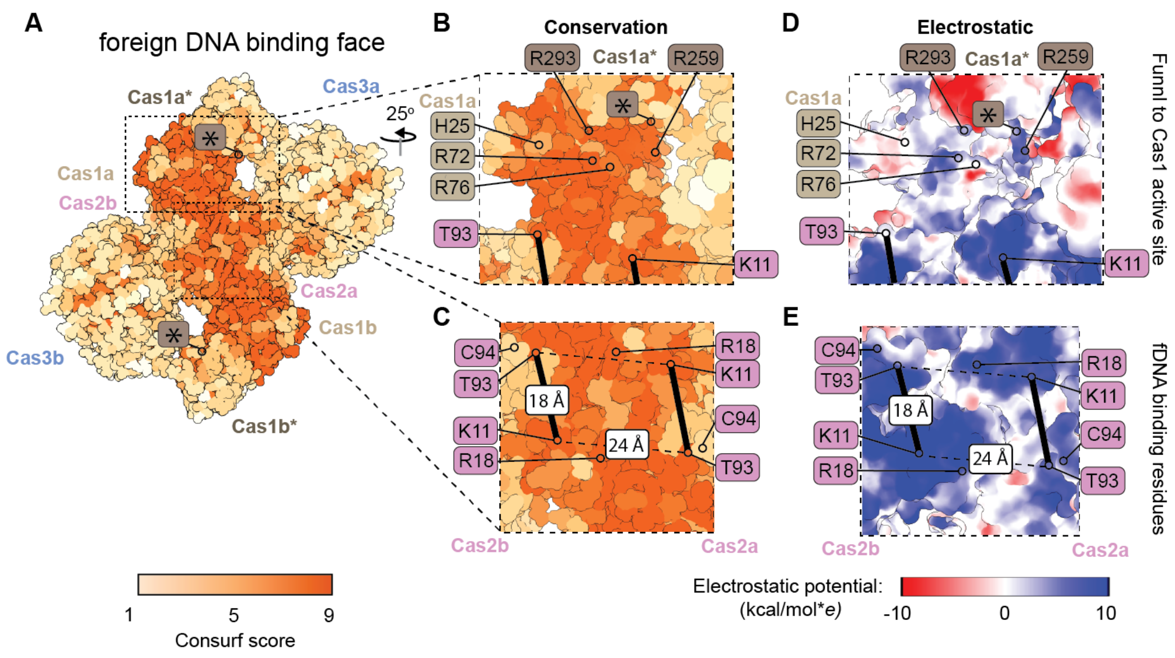

Figure S4. Positively charged foreign DNA binding residues are conserved in the Cas1-2/3 complex. Related to Figure 1. (A) Multiple sequence alignments of Cas1 (n=679) and Cas2/3 (n=745) from type I-F systems were used to calculate Consurf conservation scores, and mapped back to the atomic model of the complex (1 – least conserved, 9 – most conserved). Asterisk (*) indicates the Cas1 transesterification sites. (B, D). Conserved positively charged residues line a channel formed from the foreign DNA binding face to the Cas1 active site. Channel edges indicated with thick black lines. Distances are measured between the T93 OG1 and K11NZ using the distances command in ChimeraX. (C, E) DNA binding residues on the foreign DNA binding face of Cas2 sit in conserved patches of positive charge. Channel edges indicated with thick black lines.

bioRxiv preprint doi: https://doi.org/10.1101/2025.06.10.658980; this version posted June 11, 2025. The copyright holder for this preprint (which was not certified by peer review) is the author/funder, who has granted bioRxiv a license to display the preprint in perpetuity. It is made available under aCC-BY 4.0 International license.

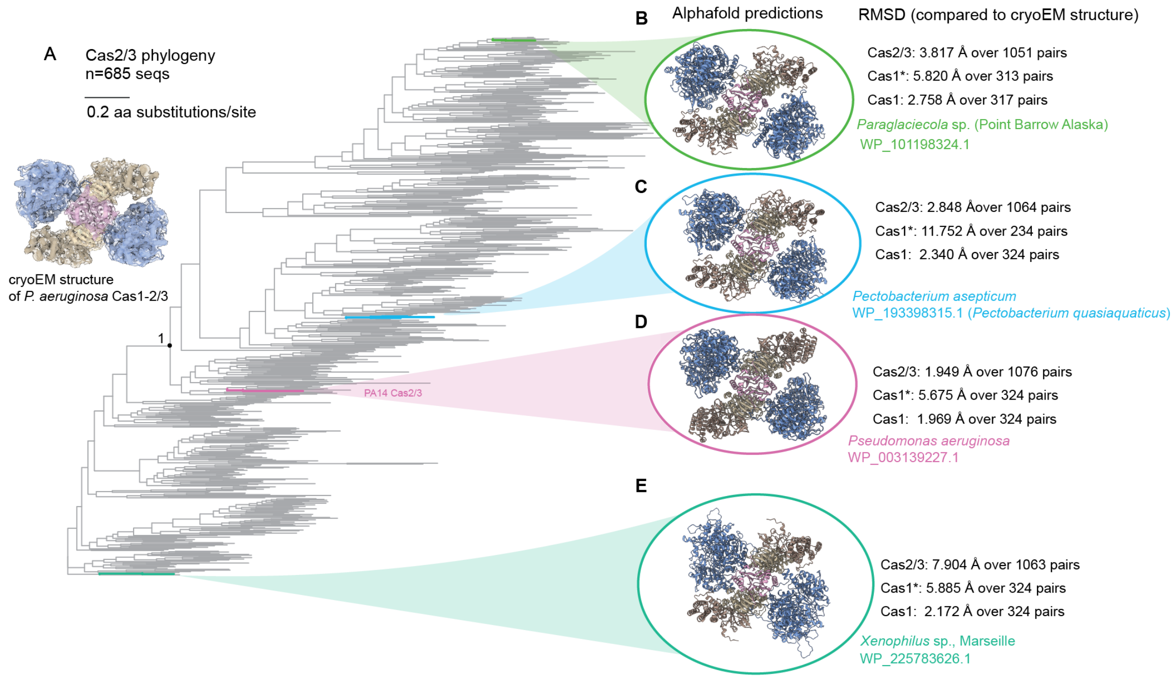

Figure S5. The four-bladed propeller shape of Cas1-2/3 systems is conserved. Related to Figure 1. (A) Phylogenetic tree of 685 Cas2/3 sequences. Sequences were identified through three iterations of PSI-BLAST, aligned in MAFFT, and the phylogenetic tree built in FastTree (see Methods). (B-E). AlphaFold3 predictions of Cas1-Cas2/3 heterohexamers reveal structural conservation. AlphaFold3 prediction were superimposed on the experimentally-determined structure using the mmaker command in ChimeraX. Root-mean square deviation of each subunit compared to across diverse type I-F systems are reported.

bioRxiv preprint doi: https://doi.org/10.1101/2025.06.10.658980; this version posted June 11, 2025. The copyright holder for this preprint (which was not certified by peer review) is the author/funder, who has granted bioRxiv a license to display the preprint in perpetuity. It is made available under aCC-BY 4.0 International license.

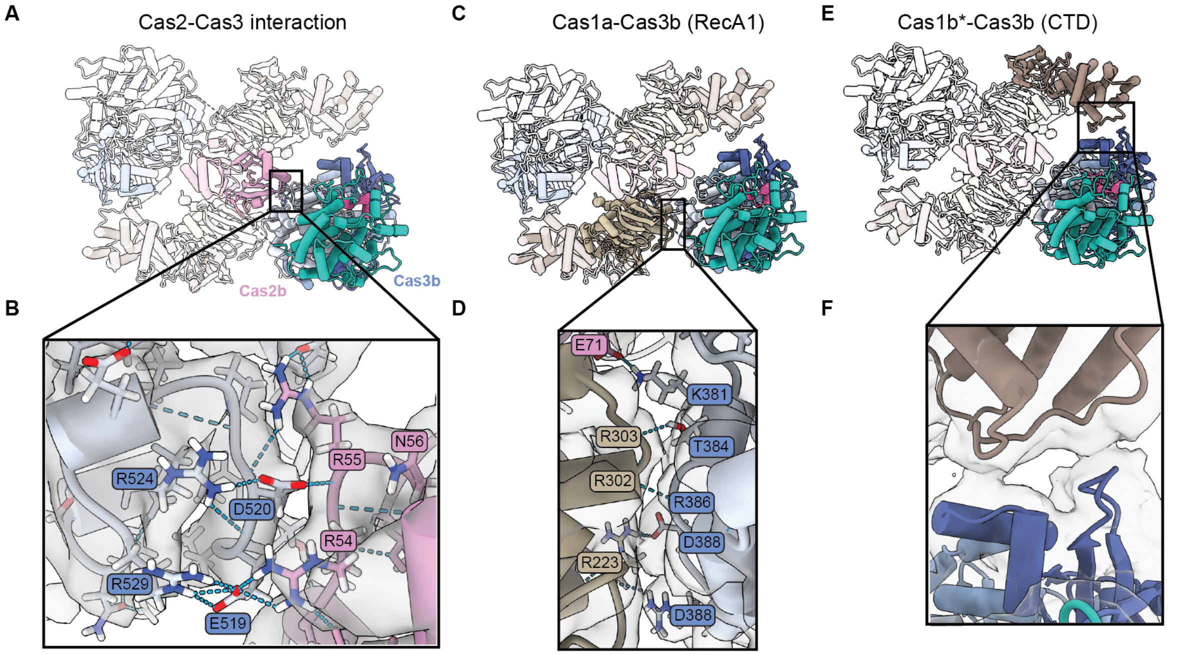

Figure S6 Three interfaces clamp Cas3 in place in the absence of DNA or Cascade. Related to Figure 1 and Figure 3. (A) Global view of the Cas1-2/3 complex, with the Cas2/3 interface responsible for blocking genome-interacting residues (R55) and locking the RecA1 domain in place shown for a single Cas2/3 subunit, all other subunits displayed at 90% transparency. (B) Hydrogen bond interactions between Cas2 (K50, R54, R55) and Cas3 (D472, D473, N373, E519 D520) reveal that the CRISPR leader interaction residues are sequestered in Cas3's RecA1 domain, density map shown at 80%, contour level = 0.1. (C) Global view of the Cas1-2/3 complex, with the Cas1-Cas3 interface in the RecA1 domain highlighted in full color, all other subunits displayed at 90% transparency. (D) Side chain and main chain hydrogen bonds at the RecA1-Cas1 interface bury ~500 Å^2. Density map shown at 80%, contour level = 0.1. (E) Global view of the Cas1-2/3 complex, with the Cas1-Cas3 CTD domain highlighted in full color, all other subunits displayed at 90% transparency. (F) Flexibility at the Cas1-Cas3 CTD interface precludes side-chain modelling, likely due to multiple transient interaction between Cas1 and the CTD Density map shown at 80%, contour level = 0.0477. AlphaFold3 predicts multiple hydrogen bonds between acidic residues of Cas3 CTD and basic residues of Cas1.

bioRxiv preprint doi: https://doi.org/10.1101/2025.06.10.658980; this version posted June 11, 2025. The copyright holder for this preprint (which was not certified by peer review) is the author/funder, who has granted bioRxiv a license to display the preprint in perpetuity. It is made available under aCC-BY 4.0 International license.

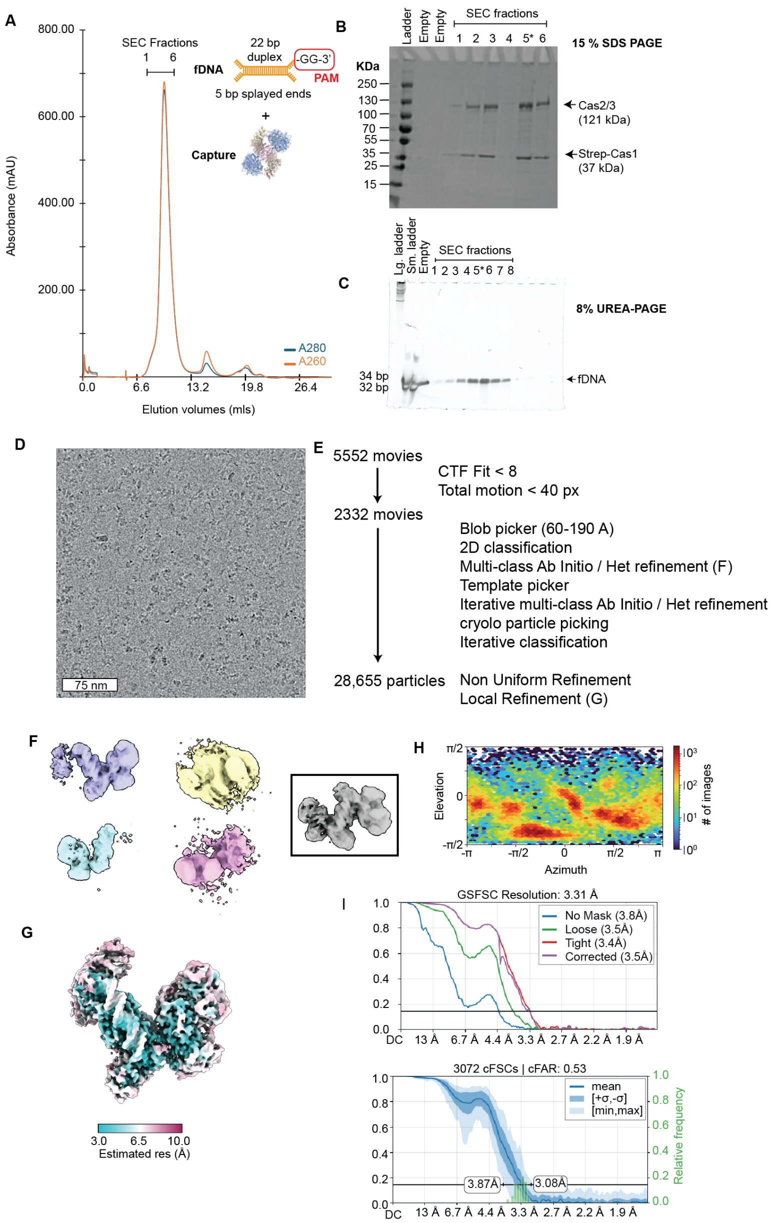

Figure S7. Purification and data processing of the Cas1-2/3 capture complex. Related to Figure 3. (A) The Cas1-2/3 complex bound to a fragment of DNA elutes from size exclusion chromatography as a monodispersed peak with an estimated molecular weight of 415 kDa. (B) SDS-PAGE stained with Coomassie blue reveals two proteins consistent with the sizes of Cas1 and Cas2/3. Asterisk (*) indicates fraction on which cryo-EM data was collected. Fraction four was lost due to spilled tube during preparation of SDS-PAGE gel (C) Urea-PAGE denaturing gel of SEC fractions confirms that the main peak contains a fragment of DNA. Asterisk (*) indicates fraction used for cryo-EM. (D) Sample micrograph at a nominal magnification of 96,000x. (E) Data processing summary. (F) Volumes from a 5-class *ab initio* reconstruction and heterogenous refinement after preliminary particle picking. The grey volume (boxed in black outline) was used subsequently as a template for template picking. (G) Local refinement of 28,655 particles colored by local resolution at estimation (FSC 0.5) reveals most of the complex is resolved below 6.5 Å but the edges of the complex are low resolution. (H) Azimuth plot reveals patchwork orientation bias, (I) FSC and conical FSC curves from cryoSPARC.

bioRxiv preprint doi: https://doi.org/10.1101/2025.06.10.658980; this version posted June 11, 2025. The copyright holder for this preprint (which was not certified by peer review) is the author/funder, who has granted bioRxiv a license to display the preprint in perpetuity. It is made available under aCC-BY 4.0 International license.

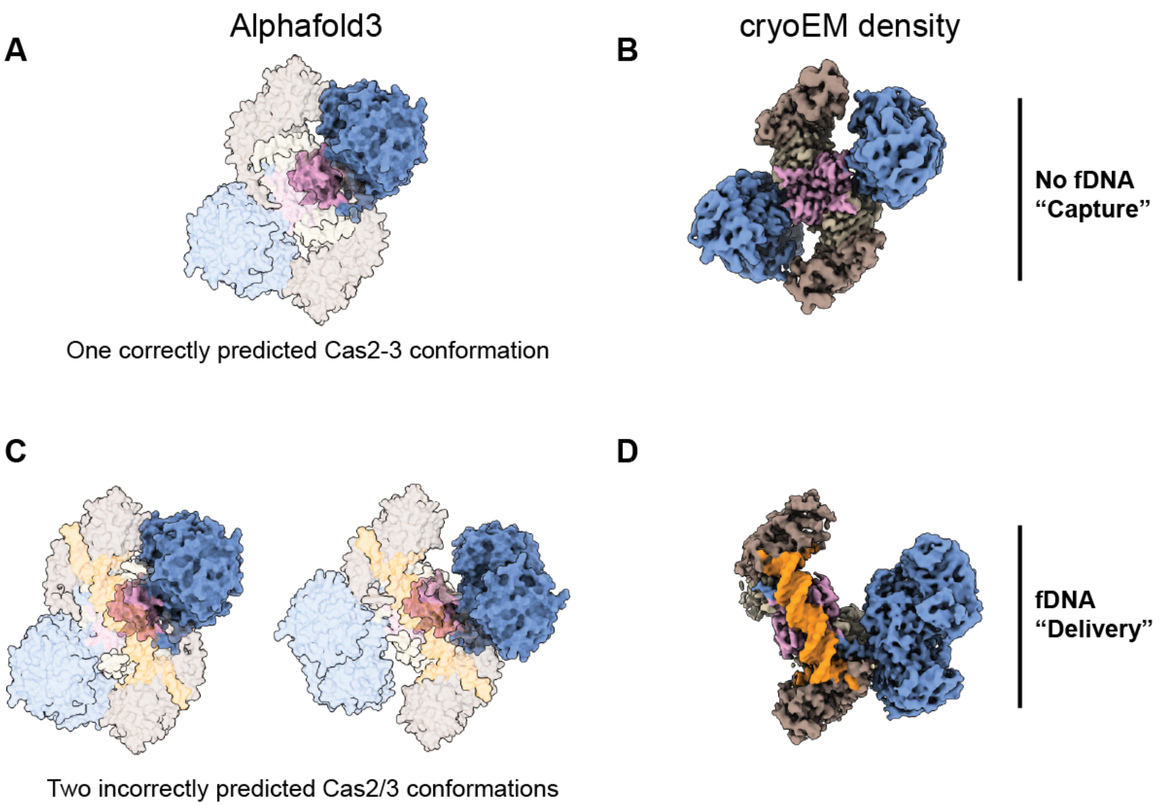

Figure S8. AlphaFold3 prediction support variable positioning of Cas3 after foreign DNA binding. Related to Figure 3. (A) AlphaFold3 prediction of the Cas1-2/3 complex bound in the absence of foreign DNA. (B) cryo-EM density for the Cas1-2/3 complex in the absence of foreign DNA agrees with AlphaFold3 prediction (map threshold=0.123). (C) AlphaFold3 predicts Cas3 in two distinct positions in the presence of a 32 bp foreign DNA fragment. (D) cryo-EM map of Cas1-2/3 bound to foreign DNA reveals one Cas3 is too flexible to resolve, while the other Cas3 domain is in an orientation that does not agree with the AlphaFold3 position (map threshold=0.195).

bioRxiv preprint doi: https://doi.org/10.1101/2025.06.10.658980; this version posted June 11, 2025. The copyright holder for this preprint (which was not certified by peer review) is the author/funder, who has granted bioRxiv a license to display the preprint in perpetuity. It is made available under aCC-BY 4.0 International license.

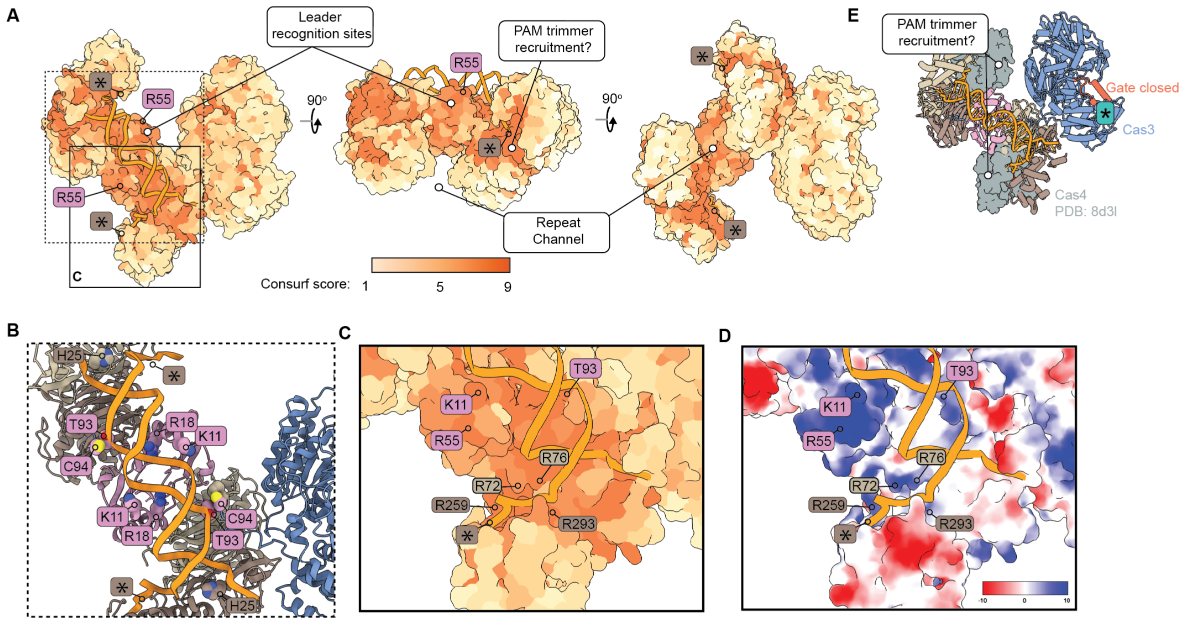

Figure S9. 3´ ssDNA channel and additional DNA binding sites are conserved. Related to Figure 3. (A) Multiple sequence alignments of Cas1 (n=679) and Cas2/3 (n=745) from type I-F systems were used to calculate Consurf conservation scores, that are mapped back to the atomic model of the Cas1-2/3 complex bound to foreign DNA (1 – least conserved is light yellow, while 9 – most conserved is dark orange). Conserved residues around the Cas1 active site are positioned to facilitate recruitment of a PAM-trimming nuclease. Asterisk (*) indicates the Cas1 transesterification sites. (B) Cas2 residues hydrogen bond with the DNA backbone to position the DNA fragment symmetrically in the foreign DNA binding channel. Asterisk (*) indicates the Cas1 transesterification sites. (C-D) The electrostatic funnel that positions the 3´ end of the foreign DNA in the Cas1 active site is conserved. Atomic model in D colored by electrostatic potential (kcal/mol*e at 298 K) in ChimeraX. (E) DNA-binding triggered conformational changes position the HD domain away from the PAM but expose faces that recruit Cas4 in other CRISPR systems. The RecA1 gate of the HD domain (colored orange) remains in the closed position after foreign DNA binding. A previously determined structure Cas1-Cas2-Cas4 (PDB: 8d3l) was docked into the foreign DNA-bound structure using the mmaker command in ChimeraX. The Cas1 and Cas2 from 8d3l were hidden and Cas4 is displayed in grey with surface representation. Asterisk (*) indicates the HD domain active site of Cas3.

bioRxiv preprint doi: https://doi.org/10.1101/2025.06.10.658980; this version posted June 11, 2025. The copyright holder for this preprint (which was not certified by peer review) is the author/funder, who has granted bioRxiv a license to display the preprint in perpetuity. It is made available under aCC-BY 4.0 International license.

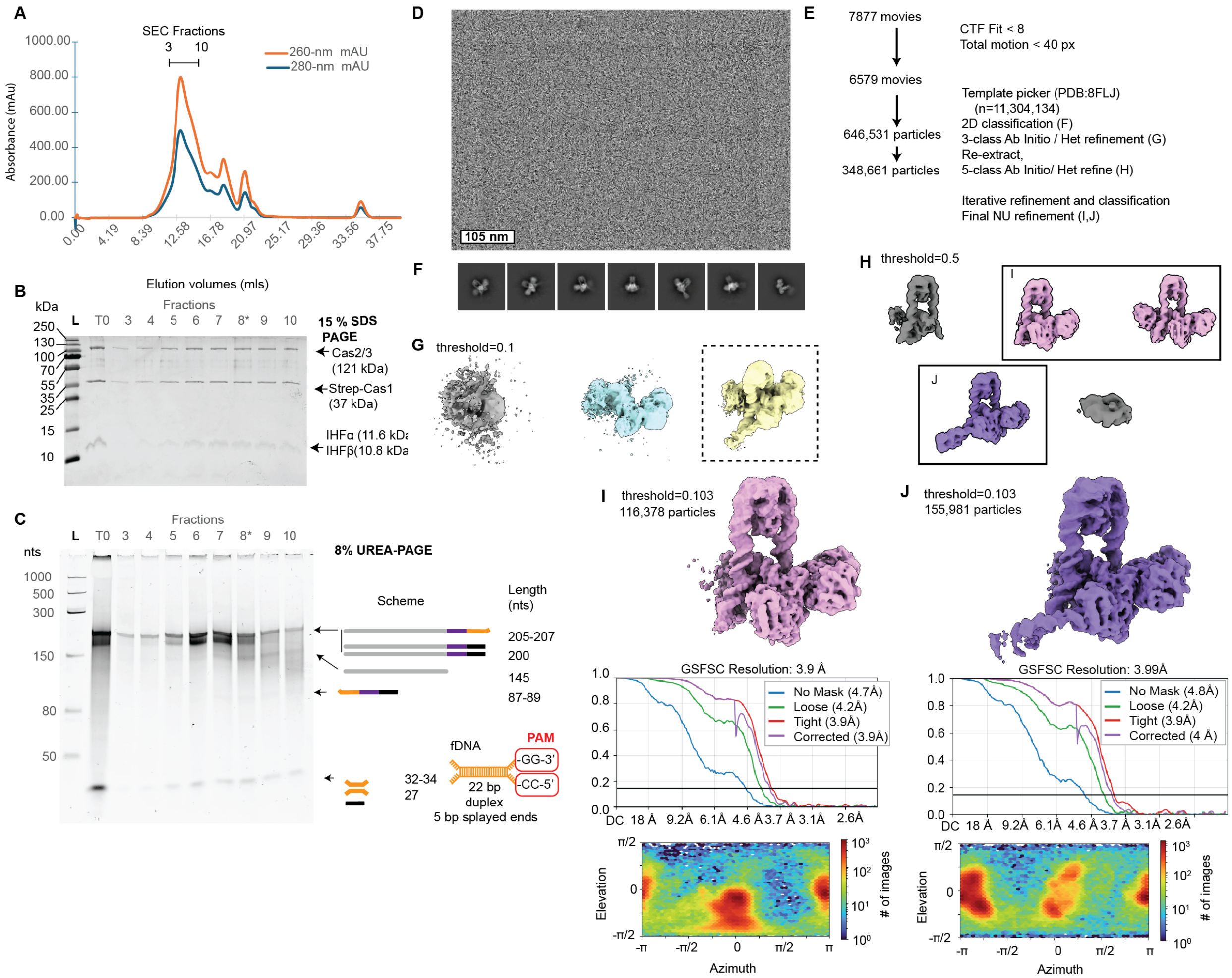

Figure S10. Purification and cryo-EM data analysis of PAM-containing integration complex. Related to Figure 4. (A) Size exclusion chromatogram of the integration reaction after 10 minutes at 25 ºC. (B) 15 % SDS PAGE gel of the main peak fractions reveals all of the protein components of the integration complex are present. Fraction 8 (green box) was frozen for cryo-EM. (C) 8 % Urea-PAGE gel of main peak fractions reveals that all of the DNA components of a partial integration reaction are present in fraction 8. (D) Sample micrograph from data collection. (E) Data analysis workflow. (F) Representative 2D class averages selected for further processing. (G) 3-class *ab initio* and Heterogeneous refinement identifies a single integration complex class (black box). (H) Five-class ab-initio reconstruction and heterogeneous refinement of integration complex particles. Black boxes indicate particle stacks used in subsequent analyses (I) Non-uniform refinement of 116,378 particles containing the U-bend but not the loop indicative of the first transesterification, FSC curve and azimuth plot displayed below (threshold 0.103). (J) Non-uniform refinement of 155,981 particles containing the U-bend and loop indicative of the first transesterification FSC curve and azimuth plot displayed below (threshold 0.103).

bioRxiv preprint doi: https://doi.org/10.1101/2025.06.10.658980; this version posted June 11, 2025. The copyright holder for this preprint (which was not certified by peer review) is the author/funder, who has granted bioRxiv a license to display the preprint in perpetuity. It is made available under aCC-BY 4.0 International license.

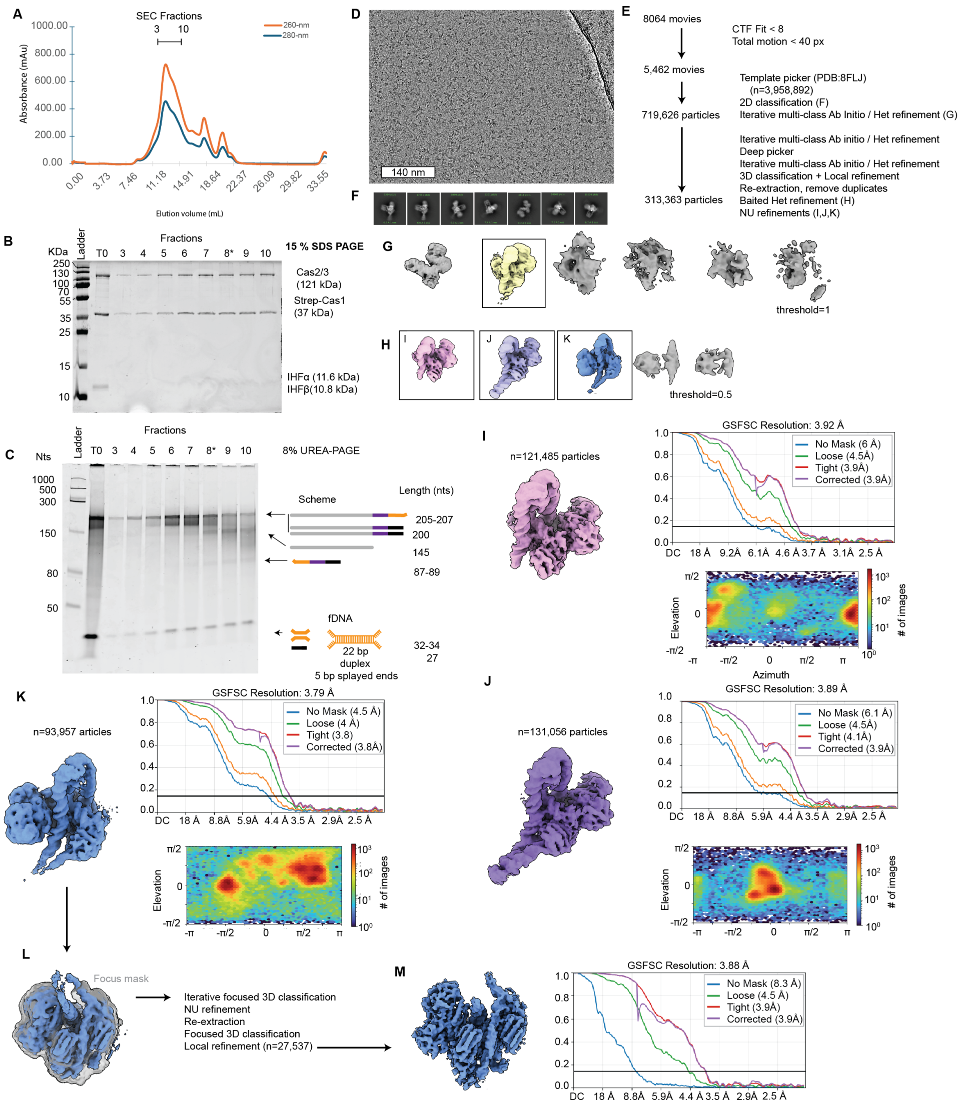

Figure S11. Purification and cryo-EM data analysis of PAM-containing integration complex. Related to Figure 4 and Figure 5. (A) Size exclusion chromatogram of the integration reaction after 10 minutes at 25 ºC. (B) 15 % SDS PAGE gel of the main peak fractions reveals all of the protein components of the integration complex are present. Fraction 8, marked with an asterick (*) was frozen for cryo-EM. (C) 8 % UREA-PAGE gel of main peak fractions reveals that all of the DNA components of a partial integration reaction are present in fraction 8. (D) Sample micrograph from data collection. (E) Data analysis workflow. (F) Representative 2D class averages selected for further processing. (G) 6-class *ab initio* and heterogeneous refinement identifies a single integration complex class (black box). (H) 6-class *ab initio* and heterogeneous refinement of the integration complex class from panel G identifies multiple distinct complexes (black outlines). (I) Non-uniform refinement, FSC curve and azimuth plot for the integration complex prior to the first integration reaction. (J) Non-uniform refinement, FSC curve and azimuth plot for the integration complex after the first integration reaction, which contains the DNA loop that is directed by IHF into the first transesterification site. (K) Non-uniform refinement, FSC curve and azimuth plot for the integration complex after the second integration reaction, when both strands of the repeat have been covalently linked to the foreign DNA.

bioRxiv preprint doi: https://doi.org/10.1101/2025.06.10.658980; this version posted June 11, 2025. The copyright holder for this preprint (which was not certified by peer review) is the author/funder, who has granted bioRxiv a license to display the preprint in perpetuity. It is made available under aCC-BY 4.0 International license.

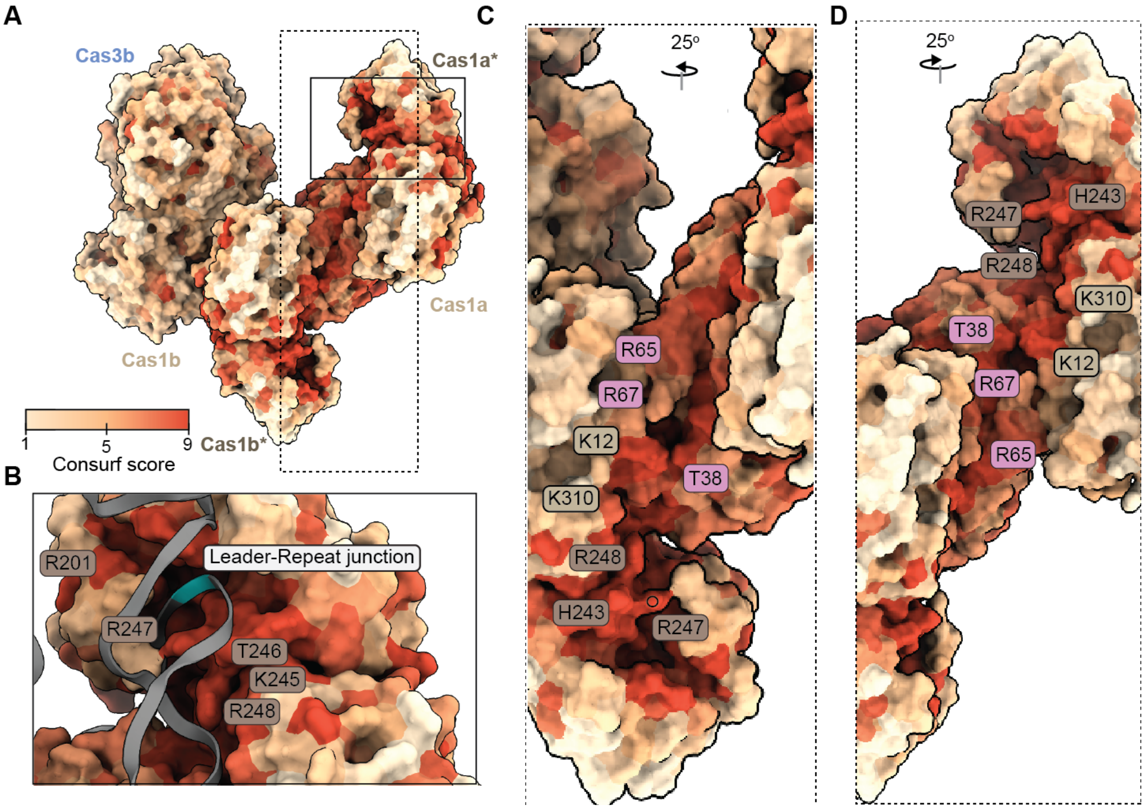

Figure S12. Positively charged residues in the repeat channel are conserved in type I-F CRISPR systems. Related to Figure 4 and Figure 5. (A) Consurf analysis of the integration complex with DNA removed to show the conserved residues of the repeat channel. (B) K245, T246, R247, and R248 are conserved and interact with the B-form portion of the CRISPR repeat in the channel just after the leader-repeat junction. (C, D) Conserved basic and polar residues line the region of the repeat channel that distorts the repeat DNA.

bioRxiv preprint doi: https://doi.org/10.1101/2025.06.10.658980; this version posted June 11, 2025. The copyright holder for this preprint (which was not certified by peer review) is the author/funder, who has granted bioRxiv a license to display the preprint in perpetuity. It is made available under aCC-BY 4.0 International license.

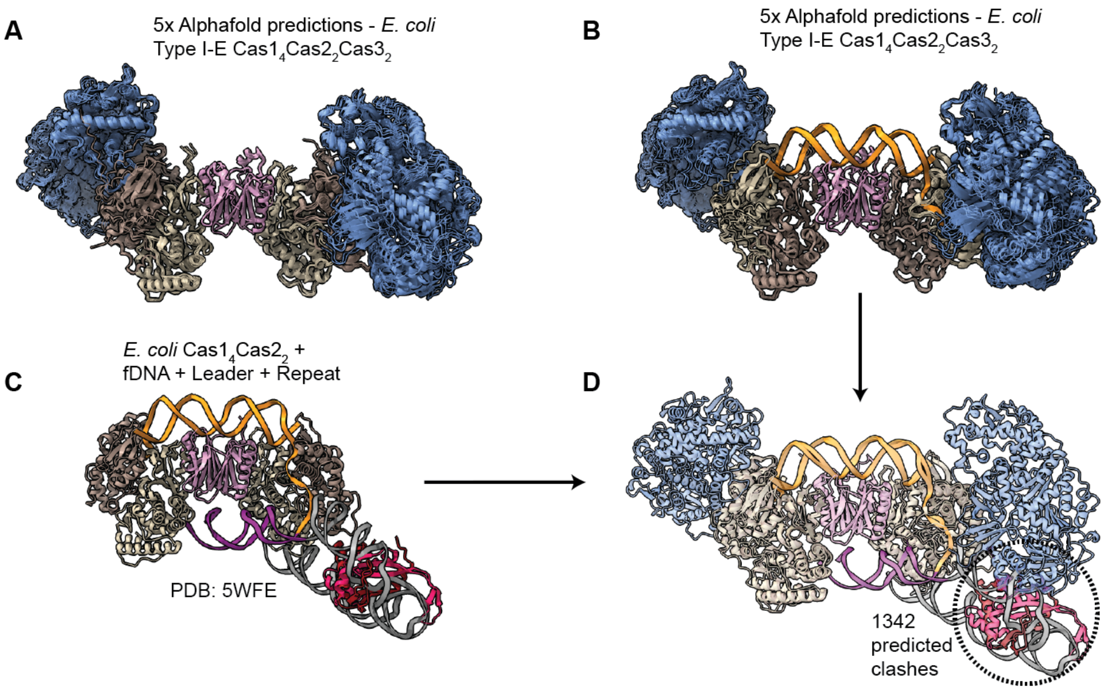

Figure S13. Cas3 in a predicted Cas1-2-3 complex from *E. coli* blocks interactions between the folded genome and Cas1. **Related to Figure 1, Figure 3, Figure 4.** (A) All five predicted complexes from an AlphaFold3 prediction of a Cas1$_4$Cas2$_2$Cas3$_2$ hetero-octamer predict Cas3 in the same conformation on the Cas1 dimer (mean iPTM across five models= 0.48). (B) Addition of a short dsDNA fragment to the prediction does not change the location of the predicted Cas1-Cas3 association (mean iPTM across five models = 0.66). (C) The Cas1-2 integration complex from *E. coli* docked onto an AlphaFold3 predicted structure of the DNA-bound Cas1$_4$Cas2$_2$Cas3$_2$ hetero-octamer reveals 1342 atomic clashes between the folded leader, which interacts with Cas1, and Cas3, which is predicted to interact with Cas1 at the
